# Patients' experiences of obstetric and gynaecological care in Québec: interactions with healthcare providers, respect of rights, satisfaction, and trust in the healthcare system

**DOI:** 10.1080/21642850.2026.2622164

**Published:** 2026-01-28

**Authors:** Sylvie Lévesque, Arianne Jean Thorn, Alexandra Toupin, Anna Medvetskaya, Isabelle Boucoiran, Natacha Godbout, Sarah Landry, Marie-Ève Blanchard

**Affiliations:** a Université du Québec à Montréal, Département de Sexologie, Montréal, Canada; b Université du Québec à Montréal, Département de Psychologie, Montréal, Canada; c Centre Hospitalier Universitaire Sainte-Justine, Montréal, Canada; d Université de Montréal, Faculté de Médecine et École de Santé Publique, Montréal, Canada; e Mouvement pour l’autonomie dans l’enfantement, Montréal, Canada; f Regroupement Naissances Respectées, Montréal, Canada

**Keywords:** Obstetric and gynaecological care, respects of rights, satisfaction with care, trust in the healthcare system, patients' experiences

## Abstract

**Background and objectives:**

Obstetric and gynaecological care profoundly affects individuals’ health and well-being. However, many international studies reveal negative and even dehumanizing experiences of this type of healthcare. This study documents recent experiences of obstetric and gynaecological care in Québec. We examine patient–healthcare provider interactions, respect for patient rights, whether patient needs are met, and how these factors impact satisfaction with the treatment provided and trust in the healthcare system.

**Methods:**

The PAROLES project is based on a self-report questionnaire posted online from July 2023 to January 2024. The sample comprises 1490 respondents who reported 1599 recent experiences of obstetric and gynaecological care. Descriptive analyses and linear regressions were performed.

**Results:**

Generally, patient–healthcare provider interactions were perceived positively, but some significant shortcomings were revealed in terms of unmet needs for shared decision-making and disrespected rights. Although patients were relatively satisfied with their care, there was considerable distrust in the healthcare system. Meeting needs in terms of shared decision-making and respecting rights were associated with overall satisfaction and trust in the healthcare system.

**Conclusion:**

The results uncover substantial inadequacies in obstetric and gynaecological care in Québec. To optimize patient–healthcare provider interactions and better meet patient needs, there must be greater respect for patients' rights.

## Background

Gynaecology practice focuses on the female reproductive system (Cunningham et al., 2014). It includes obstetric healthcare and the health and well-being of both patient and foetus through pregnancy, labour, delivery, and the postnatal period (Cunningham et al., 2014). Taken together, obstetric/gynaecological (OB-GYN) services play a vital role in the overall health of individuals. However, several studies have uncovered suboptimal care and unsatisfactory or dehumanizing experiences for patients (Bohren et al., 2015, 2019; Cárdenas-Castro & Salinero-Rates, 2023; Chancogne & Lévesque, 2024; Lévesque et al., 2016; Toupin & Lévesque, 2025; Vedam et al., 2019).

### Factors that positively influence the obstetric/gynaecological experience

Researchers have identified several factors that promote positive experiences for OB-GYN patients. More specifically, certain factors are associated with satisfaction, perceptions of high-quality service, and positive experiences of care provision such as healthcare providers’ attention to patients' concerns (Aoude, 2023; Engeltjes et al., 2023; Trainor et al., 2020; Worabo et al., 2024), respectful, nonjudgmental treatment (Bohren et al., 2020; Qaderi et al., 2021; Redshaw et al., 2019), continuous support and personalized treatment (Cai et al., 2024; Redshaw et al., 2019), respect for patients' wishes (Aranda et al., 2024; Attanasio et al., 2014; Aydin et al., 2022), and healthcare providers’ empathy (Aoude, 2023; Attanasio et al., 2014; Engeltjes et al., 2023; Qaderi et al., 2021). Moreover, when healthcare providers prioritize clear communication (Aranda et al., 2024; Attanasio et al., 2014; Aydin et al., 2022; Qaderi et al., 2021; Redshaw et al., 2019), strict confidentiality (Aranda et al., 2024; Qaderi et al., 2021), patients' physical and psychological safety (Cai et al., 2024), and patients' personal control over decisions (Aoude, 2023; McCrea & Wright, 1999), the healthcare experience tends to be more positive.

### Factors that negatively influence the obstetric/gynaecological experience

Factors that negatively affect the OB-GYN experience have also been identified. Failure to meet patients' physical and relational care needs (e.g. the need to be heard and to express one’s feelings and fears while feeling unheard, unseen, and ignored; physical separation from partners) was related to negative experiences and feelings of isolation (Trainor et al., 2020). When healthcare providers overlook patients' feelings and communicate inappropriately, notably by ignoring patients' worries and omitting to explain treatments, patients can feel dissatisfied and neglected (Akbas, 2019). Unmet needs for safety, communication, and pain management are also associated with patient dissatisfaction, and are related to high morbidity rates (Brown, 2017).

Negative experiences in connection with disrespectful care, lack of emotional support, and poor relationships with healthcare providers can exacerbate patients' feelings of helplessness and sow distrust in healthcare providers, which in turn affects patients' future health-seeking behaviour (Vargas et al., 2021). Perceptions of maltreatment, incompetence, and discrimination in the healthcare experience continue to feed patients' fears and mistrust, even after recent healthcare experiences that were generally positive (Prall et al., 2024).

### Prevalence—studies on satisfaction and dissatisfaction with healthcare

Patients' satisfaction with OB-GYN care and the quality of their interactions with healthcare providers are important indicators for evaluating and improving healthcare services. Across various studies conducted worldwide, the majority of patients appear satisfied with the services received. A study conducted among 246 patients in an institution’s obstetrics/gynaecology clinics in the United States found that 91% of participants felt listened to and 84% expressed satisfaction with treatment discussion (Bhuiyan & Loder, 2024). They also reported feeling respected and comforted during physical exams (96%). However, studies have shed light on various disparities in different contexts, and documented sources of dissatisfaction often relate to respect for privacy, communication with healthcare professionals, access to information regarding their rights or care, and respectful attitudes toward patients (Birhanu et al., 2010; Kowalewska et al., 2014). A study conducted in France among 627 women attending one of 25 maternity units found that over 11% reported dissatisfaction due to disrespectful behavior from healthcare providers, most often involving disregarding women’s pain or excluding them from decision-making (Gaucher et al., 2021).

### Healthcare in Canada and Québec

Canada’s healthcare system is based on the Canada Health Act, which provides for universal insured health services (Government of Canada, Library of Parliament, 2019). Although each province and territory administers its own healthcare system, they must all conform with certain basic national principles: public administration (administration and operation on a nonprofit basis by a public authority), comprehensiveness (insurance coverage for all medically necessary services provided by diverse entities), universality (equal access for all residents), portability (continued coverage for travel within Canada), and accessibility (access to medically necessary services based on need and not ability to pay) (Government of Canada, 2024). Like all the other provinces, Québec has its own particularities. Although the majority of costs for obstetric/gynaecological care are covered by the *Régime de l’assurance maladie du Québec* (RAMQ; Québec’s health insurance plan^1^) some population groups are excluded (e.g. individuals waiting for official Québec status, certain individuals with internship or working permits). Moreover, some sexual health services, such as contraception, are partially or entirely paid services, which limits access (Di Meglio & Yorke, 2019). For obstetric care, Québec offers diverse forms of pregnancy follow-up (with obstetrician/gynaecologists, family doctors, or midwives) and birthing facilities (hospital, birthing house, home) (Gouvernement du Québec, 2018), but access to these services remains unequally distributed across the province’s regions, with medical deserts in rural and remote areas (Gauthier et al., 2009).

Although Québec has a socially and culturally diverse population, it faces challenges in terms of its trauma-informed care and intercultural approach, notably due to lack of training for care providers to support diversified pathways. This impacts individuals who are, among others, transgender, nonbinary, disabled, racialized, immigrants, or First Nations (Adams et al., 2024; Ghabrial et al., 2024; Scheim et al., 2021; Wylie et al., 2021).

Empirical data on the experience of reproductive health care from the situated point of view of those who receive it is lacking in Quebec. How obstetric and gynaecological care is carried out, participation and decision-making autonomy, obtaining consent and respecting patients' rights are aspects of care that are poorly documented. Therefore, the lack of quantitative data in Québec limits the understanding of how patients experience healthcare provision and the impacts on their satisfaction with obstetric/gynaecological care. The PAROLES project aims to fill this gap by gathering data on the personal experiences of obstetrics/gynaecology patients.

## Objectives

Based on patients' reports of recent healthcare experiences, this study had the following objectives: 1) document patients' experiences of interactions with healthcare providers and the respect of their rights in the area of obstetric and gynecological care; 2) evaluate the extent to which the healthcare that patients received met their needs; and 3) determine levels of patient satisfaction and trust in Québec’s healthcare system following a recent obstetric or gynecological healthcare experience.

### Theoretical framework

The conceptual framework chosen for this project integrates two models: the Montreal Model and the Theory of Patient-Centered Communication. The Montreal Model (Cote, 2024; Flora et al., 2015), also known as the patient-partner model, is grounded in four core components. This model emphasizes the value of patients' experiential knowledge, which is recognized as an essential complement to the scientific expertise of healthcare professionals. The synergy between experiential and scientific knowledge enables care that is better adapted to patients' needs. The care relationship (or care management) is approached as a co-construction, in which both the patient and healthcare professionals engage in dialogue and collaboratively develop care decisions. This necessitates a sharing of power, as well as a reframed concept of responsibility, whereby the patient is not viewed as a passive recipient of care but as an engaged and agentive partner. Finally, the model operates on multiple levels; it is not limited to the therapeutic relationship itself, but also encompasses all care-related activities and structures, spanning organizational and systemic domains. The Theory of Patient-Centered Communication (Epstein et al., 2005) posits that effective medical communication prioritizes the patient’s perspectives, values, and needs. This theory also highlights the importance of the patient’s active participation in health-related decisions, particularly those concerning treatment goals. Within this dual conceptual framework, clinicians are encouraged to adopt an empathic stance that fosters trust and emphasizes the patient–professional relationship, with the aim of sharing both power and responsibility. This theoretical framework is therefore relevant to our study, as we seek to document patients' perspectives regarding the respect of their rights and needs. In addition, we assess the quality of shared decision-making between patients and healthcare professionals regarding the treatment plan and recommendations, a central principle both of the Patient-Centered Communication theory and the Montreal model. Finally, we take into consideration the interpersonal skills of the professional, essential for understanding not only patients' level of satisfaction with their healthcare experience, but also their overall trust in Québec’s healthcare system.

Our research objectives include several concepts that we define here for consistent results interpretation. Experiences are understood as what individual patients undergo while receiving healthcare. In this study, we focus on interactions with care providers and care providers’ respect of patient rights. Thus, the participants described specific incidents (e.g. whether a right was transgressed) that occurred when they received healthcare. Needs refer here to patients' expectations or preferences in connection with their healthcare experiences. These needs are measured in terms of the subjective importance the participants placed on various aspects of the experience. Interactions refer to discussions and behavioural interactions between patients and care providers as observed and perceived by the patients (e.g. do care providers explain medical exam results?). Rights are understood as basic and inalienable guarantees for individuals during healthcare access and provision, as set forth in legal frameworks. In the present study, they include the rights to information, consent, respect for dignity and intimacy, non-discrimination, and autonomy. Patient satisfaction refers to the patient’s subjective evaluation of the overall reported healthcare experience. It is associated with several dimensions, including trust in the healthcare system and perceptions of healthcare quality and the interpersonal skills and competence of healthcare providers.

## Materials and methods

### The PAROLES project

The PAROLES project was the outcome of the work of a research partnership team that brought together researchers and representatives of community organizations and professional associations. It was funded as part of a concerted action by the *Fonds de recherche du Québec—Société et Culture* (FRQ-SC; Québec research fund—society and culture) and the *Secrétariat à la condition feminine* (Secretariat for women’s affairs). As part of a three-phase project, this article addresses the first phase: a quantitative study of self-reported experiences of OB-GYN care.

We conducted the study in several steps. First, we reviewed the literature to compile a list of the various dimensions of obstetric/gynaecological care. We performed a second review to identify tools to measure obstetric/gynaecological care, satisfaction with care provision, and obstetric/gynaecological violence. In this step, we also obtained authorization by the *Birth Place Lab*, whose Principal Investigator, Saraswathi Vedam, is collaborator on the PAROLES project, to use measurement tools developed in the Canadian RESPCCT Study. Several discussions were held with researchers from other countries with expertise in OB-GYN violence. Through these exchanges, we acquired tools and recommendations for our study. Finally, questionnaire items were developed in French for each dimension of interest, most of which were adapted from the RESPCCT Study (Vedam et al., 2017; Vedam et al., 2019).

The PAROLES project team drew on the expertise of research partners from community groups (e.g. an organization for the defence of perinatal rights, a network of immigrant and racialized women, a disabled persons defense group) and institutional groups (e.g. *Ordre des infirmières et infirmiers du Québec* [Québec order of nurses], *Collège des Médecins* [College of physicians], *Ordre des Sages-Femmes du Québec* [Québec order of midwives). These partners advised us on the relevance of our proposed items, the absence of certain essential elements, and items to prioritize, which helped us identify key areas to address in the Québec context. The team then presented the questionnaire to individuals with varying profiles who had received obstetric/gynaecological care in Québec, and the content was subsequently reworked to incorporate their comments. The questionnaire was translated into English and made available in both English and French.

The questionnaire was designed to allow participants to respond in light of either a recent experience of obstetric/gynaecological care (i.e. the last time they received care) or else a particularly memorable experience of obstetric/gynaecological care that occurred in the part 7 years After responding to the items for the selected experience category (recent or memorable), participants could respond again by selecting the other category. Thus, they could report more than one healthcare experience. This study focuses on the first category: recent experiences.

A structured outreach strategy developed in collaboration with a public communications firm was implemented: several television, media and radio stations announced the study and explained how to participate. Participants were also recruited through partner and community organizations, who relayed the call for participation to their members, contacts, and social media networks. Finally, social media (Facebook and Instagram) were also used to convey the invitation to participate in a survey. The inclusion criteria were aged 18 years and older and having received OB-GYN care in the last seven years in Québec. Thus, cisgender women, trans men, and nonbinary individuals could participate. The self-report questionnaire was posted online on the Qualtrics platform from July 2023 to January 2024. Participants had to read the information and consent form and sign digitally before responding. Given the sensitive nature of certain questions, a list of psychosocial resources was provided. In all, 20 digital gift cards worth $50 each were drawn by lot by the participants.

### Measures

#### Sociodemographic variables

The questionnaire began with sociodemographic items to gather information on participants’ age (at the time of questionnaire completion), country of birth, sexual identity, sexual orientation, highest education level completed, and economic situation.

#### Shared decision-making

Seven items adapted from the RESPCCT Study - in British Columbia, Canada - (Vedam et al., 2017) addressed interactions with healthcare providers. Participants indicated their degree of agreement with each item (e.g. ‘They explained my test and exam results’) on a six-point Likert scale (Completely disagree = 0; Disagree = 1; Disagree somewhat = 2; Agree somewhat = 3; Agree = 4; Completely agree = 5) to rate their experience of the healthcare provision. Shared decision-making scores were calculated from the sum of each item. Higher scores indicated greater shared decision-making. For the descriptive analysis presentation, responses were dichotomized: Disagree (0–1–2 = 0) or Agree (3–4–5 = 1). Since this scale was adapted from a previous project, we conducted an exploratory factor analysis to ensure that the adapted items measured the same dimension. According to Kaiser criteria (eigenvalue > 1), all items showed good factor loadings on one factor, supporting a unidimensional structure (loadings ranging from .75 to .86) (Kaiser, 1960). This scale also showed good internal consistency (*α* = .81; *ω* = .80). Inspired by Keshet Korem, a PhD Candidate at the University of Haifa, we asked participants to rate the importance they attributed to each of the items to which they responded ‘Disagree’ in order to assess individual needs in relation to the interactions. Responses ranged from Not important = 0 to Extremely important = 5. For the descriptive analysis presentation, responses were dichotomized: Not important (0–1 = 0) or Important (2–3–4–5 = 1). When participants disagreed with an item but rated it as important, it was considered an unmet shared decision-making need. Table 2 presents descriptive data for each item.

#### Disrespect of rights

To assess whether healthcare providers respected participants’ rights, we borrowed from a scale developed for the RESPCCT Study that measures respect for confidentiality and consent (Vedam et al., 2019). Although the laws for patient rights in Québec differ slightly from those in British Columbia, because they fall under separate provincial jurisdictions, the rights to confidentiality and consent are the same for both provinces. Participants responded to eight items with one of three choices (No = 0; Yes = 1; Does not apply) (see Table 3). Two sample items are: ‘My confidential and personal information was disclosed without my consent,’ and ‘The healthcare providers asked for my permission before doing a vaginal exam.’ An exploratory factor analysis was conducted since the items were adapted. It showed two factors with eigenvalues greater than 1. The first contained 5 items and reflects the construct of *Violations of patient rights and autonomy* (factor loadings ranging from .58 to .83), while the second contained 3 items and illustrates the construct of *Provider consent-seeking behavior* (factor loadings ranging from .71 to .89). Both subscales showed acceptable internal consistency (*Violations of patient rights and autonomy subscale*: *α* = .73; *ω* = .74; *Provider consent-seeking behavior subscale*: *α* = .79; *ω* = .75). A score for each subscale based on the sum of each item was calculated. The *Provider consent-seeking behavior subscale* items were reversed, so a higher score meant more rights were not respected, as the first subscale.

#### Satisfaction and trust

Satisfaction with the care received was assessed in terms of perceived quality of care, perceived competence of healthcare providers, and patients' trust in the Québec healthcare system following the reported care. A sample item is: ‘After your last obstetric/gynaecological consultation, how would you rate the overall quality of the care you received?’ Participants rated four items on a six-point Likert scale from Completely dissatisfied (0) to Completely satisfied (5). For the descriptive analysis presentation, responses were dichotomized: Dissatisfactory (0–1 = 0) or Satisfactory (2–3–4–5 = 1). The factor loadings, derived from exploratory factor analysis, ranged from 0.75 to 0.91, supporting the unifactorial structure of the instrument.

### Analysis plan

Descriptive analyses were initially run using SPSS (dichotomized items), followed by four linear regressions (quality of care; healthcare providers’ interpersonal skills; healthcare providers’ competence; trust in Québec’s healthcare system) to assess whether interactions with healthcare providers and disrespect of rights influenced patients' satisfaction with the care they received (continuous score). For the linear regressions, we used the continuous scores for each studied dimension. The effect size was calculated using the R^2^ (coefficient of determination), i.e. the percentage of the dependent variable explained by the linear model. According to Cohen’s (Cohen, 1988) criteria, an R^2^ of 0.2 indicates a small size effect, while 0.5 and 0.8 indicate a medium and large size effect, respectively.

### Ethics statement

The PAROLES project obtained ethics approval from the ethical research committee of the first author’s university (CIEREH, No. 2023-5345).

## Results

### Participant description

**Figure 1.**
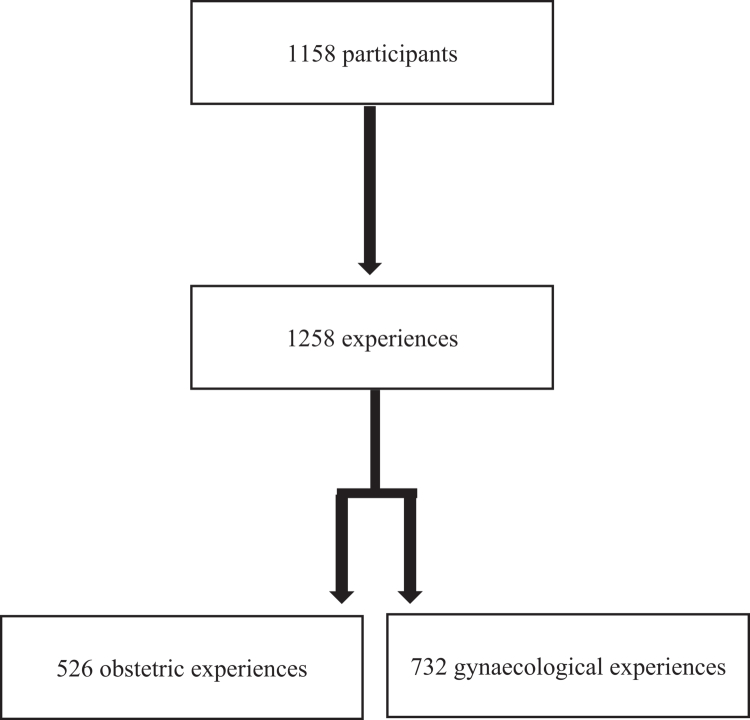
Sample composition.

The research team cleaned the data obtained from 1712 compiled questionnaires. Questionnaires containing sociodemographic data only (*n* = 185), non-serious (*n* = 10), incomplete (*n* = 338), or duplicate (*n* = 14) entries were removed from the data set. An emphasis was placed on excluding any responses that may have been provided by bots by checking completion time (treated in the previous step as part of incomplete responses), response patterns (*n* = 1) and open-ended responses (*n* = 6). The final sample comprised 1158 individuals who reported a total of 1258 recent healthcare experiences, including 526 obstetric and 732 gynaecological. The reported experiences had to have occurred within the past seven years, including the year of data collection, which spanned two years. Therefore, the reported experiences ranged from 2016 to 2023, the majority of which took place between 2020 and 2023 (78.3%). Most of the 1158 participants who reported recent healthcare experiences were aged from 26 to 35 years (51%), were born in Canada (90%), self-identified as women (94%), and reported heterosexual orientation (83%). Figure 1 presents a flowchart explaining the composition of the sample. Table 1 presents the sociodemographic characteristics.

**Table 1.** Sample characteristics (*N* = 1158 individuals).

Variable	*n*	%
**Age (years)**		
18 to 25	127	11.0
26 to 35	591	51.0
36 to 45	286	24.7
46 to 55	104	9.0
56+	50	4.3
**Gender**		
Woman	1093	94.4
Man	35	3.0
Non-binary	11	0.9
Genderfluid	4	0.3
Agender	2	0.2
Two-spirited	3	0.3
Questioning	5	0.4
**Sexual orientation**		
Heterosexual	964	83.2
Homosexual	25	2.2
Bisexual	77	6.6
Asexual	7	0.6
Pansexual	58	5.0
Queer	9	0.8
Questioning	16	1.4
**Place of birth**		
Canada	1039	89.7
Outside of Canada	119	10.3
**Education**		
Primary school	5	0.4
High school	71	6.1
College	332	28.7
Undergraduate university studies	435	37.6
Graduate university studies	315	27.2
**Perception of economic situation**		
Financially comfortable	334	28.8
Sufficient (to meet basic needs or those of the family)	731	63.1
Insufficient (to meet basic needs or those of the family)	93	8.0
***n*** **obstetric participants**	526	41.8
***n*** **gynaecological participants**	732	58.2

### Interactions with healthcare providers and unmet needs for shared decision-making

Of the 1258 reports of recent care received, about 75% involved healthcare providers who checked to see that the participants understood what was going to happen to them. About as many involved the following healthcare situations: healthcare providers informed them about medical procedures; healthcare providers were open and honest with them; and healthcare providers explained their test results (see Table 2). In contrast, only half of the 1367 experiences involved healthcare providers who informed patients that they could refuse the intervention, with about the same proportion for situations where patients felt they could question or challenge the healthcare providers’ recommendations.

**Table 2.** Shared decision-making experiences and needs (*n* = 1258 experiences).

	Recent care		
Item	Agree % (*n*^1^)	Disagree % (*n*^1^)	Disagree, but important % (*n*^1^)
The healthcare provider explained my treatment plan to me openly and transparently.	79.6 (917)	20.4 (235)	89.3 (209)
They explained my treatment procedure.	77.0 (932)	23.0 (278)	90.6 (251)
They took the time to make sure that I understood what was happening to me.	74.7 (906)	25.3 (307)	94.4 (289)
They always made sure that I was aware that I could refuse an exam or a procedure.	51.3 (585)	48.7 (556)	85.9 (476)
They explained my test or exam results to me.	75.0 (826)	25.0 (275)	90.9 (250)
I felt that I could question the decisions and recommendations of the healthcare provider(s).	49.7 (567)	50.3 (574)	87.7 (501)

Note: ^1^ *n* does not add up to 1258 due to participants answering ‘non-applicable’.

As mentioned above, when participants disagreed with an item that was rated as important, we considered it an unmet need in shared decision-making. The proportions of unmet needs were similar across items. When a given care interaction was not delivered by healthcare providers, it was rated as important—and was therefore as an unmet need—in about 90% of cases. For example, 556 (49%) of participants disagreed with the statement: ‘They always made sure that I knew that I could refuse an exam or a procedure.’ Of these, 476 (86%) participants rated this care interaction as important, indicating an unmet shared decision-making need.

### Disrespect of rights

In over half (52%) the reports of care, at least one patient right was disrespected. For *Violations of patient rights and autonomy subscale*, 17% of individuals reported at least one right not being respected, while for the items on the *Provider consent-seeking behavior subscale*, more than half of the sample (55%) reported that the healthcare professional did not ask for prior consent. Table 3 presents the items on respect of rights, when applicable to the reported experiences. We observe that the most often transgressed rights are the right to consent to a vaginal exam (28% of transgressions), to consent to the presence of observers (40%), and to consent to have medical students perform medical procedures (45%). The most often respected rights are to the protection of personal information (93%), intimacy and dignity in connection with nudity (93%), prior consent to sterilization (96%), prior consent to treatment (88%), and the freedom to sign legal documents without restriction and in full knowledge of the facts (93%).

**Table 3.** Disrespect of rights.

	Recent care (*n*^1^ = 1258 experiences)		
Item	No % (*n*)	Yes % (*n*)	*n* (Applicable)
*Violations of patient rights and autonomy subscale*			
My confidential and personal information was disclosed without my consent.	93.3 (1024)	6.7 (73)	1097
The healthcare providers entered the room and undressed me without asking me first.	93.0 (1066)	7.0 (80)	1198
They performed surgery on me without my consent so that I can’t have any more children (sterilization).	95.9 (909)	4.1 (39)	992
I was forced to sign legal documents saying that I consented to procedures that I didn’t want or that weren’t explained to me.	93.0 (919)	7.0 (69)	1036
I was forced to accept treatment or procedures (other than sterilization) that I didn’t want.	88.4 (879)	11.6 (115)	1041
*Provider consent-seeking behavior subscale*			
The healthcare providers asked for my permission for other people (such as students or observers) to assist with my treatment.	60.0 (305)	40.0 (457)	791
The healthcare providers asked for my permission for a student to examine me or to perform procedures on me.	45.3 (369)	54.7 (306)	702
The healthcare providers asked for my permission before doing a vaginal exam.	27.8 (322)	72.2 (836)	1212

Note: ^1^ *n* may not sum up to 1258 due to participants answering ‘non-applicable’.

### Satisfaction and trust

When asked about their most recent experience of obstetric or gynaecological care, the participants rated three out of four experiences as satisfactory in terms of quality of care, with about the same ratings for the interpersonal skills of healthcare providers. Satisfaction with healthcare providers’ competence exceeded 80% (see Table 4). Nevertheless, of the participants who reported recent care, only 62% trusted Québec’s healthcare system after the experience (see Table 5).

**Table 4.** Satisfaction with care received and healthcare providers (*n* = 1258).

After your last obstetrics or gynaecology consultation, how would you rate the following overall?	Satisfaction level	
Item	Dissatisfactory % (*n*^1^)	Satisfactory % (*n*^1^)
The quality of the care you received	25.2 (317)	74.8 (940)
The interpersonal skills of the healthcare providers (e.g. willingness to listen)	24.7 (309)	75.3 (944)
The competence of the healthcare providers	16.6 (208)	83.4 (1047)

Note: ^1^ *n* does not add up to 1258 due to missing data.

**Table 5.** Trust in Québec’s healthcare system (*n* = 1258).

After your last obstetrics or gynaecology consultation, how would you rate the following overall?	Trust level		
Item	Distrust % (*n*^1^)	Trust % (*n*^1^)	Neutral % (*n*^1^)
Your trust in Québec’s healthcare system	23.1 (291)	62.2 (185)	14.7 (782)

Note: ^1^ *n* does not sum up to 1258 due to missing data.

### Associations between treatment received, satisfaction, and trust

The linear regression results show that shared decision-making and disrespected rights significantly predict satisfaction for all four items. Even if it was significant with the three other items, the prior consent for *Provider consent-seeking behavior subscale* was not significantly associated with trust in healthcare system. The four models explained from 22% to 44% of the variance in satisfaction (see Table 6). Overall, the more the decision-making was shared, the greater the satisfaction, and the more that rights were disrespected or when prior consent was not obtained, the lower was the satisfaction. These results are statistically significant for all four items: quality of care, *F*(2,815) = 127.05, *p* < .001; interpersonal skills of healthcare providers, *F*(2,815) = 157.94, *p* < .001; competence of healthcare providers, *F*(2,815) = 103.11, *p* < .001; and trust in Québec’s healthcare system, *F*(2,815) = 56.40, *p* < .001. Table 6 presents the results.

**Table 6.** Linear regression for satisfaction with shared decision-making and disrespected of rights (*n* = 818 experiences).

			95% CI				
	Estimate	*SE*	LL	UL	*p*	*R* *^2^*	*p*
**Quality of care received**						.39	<.001
Shared decision-making	.451	.029	.394	.508	<.001		
Violations of patient rights and autonomy subscale	−.184	.055	−.292	−.077	<.001		
Provider consent-seeking behavior subscale	−.118	.047	−.211	−.026	.012		
**Interpersonal skills of healthcare providers (e.g. willingness to listen)**						.44	<.001
Shared decision-making	.472	.028	.417	.526	<.001		
Violations of patient rights and autonomy subscale	−.171	.052	−.274	−.069	.001		
Provider consent-seeking behavior subscale	−.161	.045	−.249	−.073	<.001		
**Competence of healthcare providers**						.34	<.001
Shared decision-making	.302	.026	.251	.352	<.001		
Violations of patient rights and autonomy subscale	−.293	.049	−.388	−.197	<.001		
Provider consent-seeking behavior subscale	−.168	.042	−.250	−.086	<.001		
**Level of trust in Québec’s healthcare system**						.22	<.001
Shared decision-making	.344	.033	.280	.408	<.001		
Violations of patient rights and autonomy subscale	−.154	.062	−.275	−.033	.013		
Provider consent-seeking behavior subscale	−.056	.053	−.160	.048	.293		

Note: *n* does not add up to 818 due to missing data on at least one variable of interest.

## Discussion

To document patients' experiences of obstetric and gynaecological care, we examined patient–healthcare provider interactions, the alignment between these interactions and patients' needs, respect of patients' rights, and patients' satisfaction and trust in Québec’s healthcare system.

The results shed light on some worrisome dynamics in the quality of interactions between patients and healthcare providers and in the respect of patient rights. Although the majority of the reported interactions were marked by transparent communication and appropriate interpersonal skills on the part of healthcare providers, a significant portion of the provided care did not meet patients' needs or respect their rights, such as the right to refuse treatment. Despite relatively greater respect of the rights to confidentiality, intimacy, and consent to major procedures, we note that more than one out of ten participants reported being forced to accept a treatment or procedure (other than sterilization) that they didn’t want. Importantly, patient consent was not systematically sought or obtained for vaginal exams, the presence of observers, or the participation of medical interns or students. The results also show the impact of needs that are met and rights that are respected on patients' satisfaction with their care and trust in Québec’s healthcare system. Thus, although the majority of participants rated their experiences as satisfactory, only 62% indicated that they trusted the system after their healthcare experience.

Our findings echo concerns identified in works addressing informed consent and patients' rights in obstetric/gynaecological care. For example, other studies emphasized that explicit consent is essential for all invasive obstetrical procedures, and that the omission of consent is associated with psychosocial consequences such as birth-related trauma (Shalowitz & Ralston, 2023). Similarly, Dixon-Woods et al. described how power disparities and disrespect for patient autonomy can result in patient perceptions of coercive or incompletely informed consent, which foments distrust of healthcare systems (Dixon-Woods et al., 2006). These results corroborate the importance of the patient-partner model, which argues that the patient should be at the center of all healthcare (Cote, 2024; Flora et al., 2015). This model is based on power sharing, where the patient's experiences are respected and valued, which helps to build a relationship of trust not only with the healthcare professional, but also, in the long term, with the healthcare system as a whole.

Our results enrich the growing body of research on how violations of patients' rights impair weaken their satisfaction with and trust in healthcare systems. Mistrust can have negative effects in turn, such as nonadherence to medical recommendations and lower use of medications and healthcare services (Haywood et al., 2014). Studies focusing on racial discrimination have revealed that patients from racialized and minority ethnic groups frequently mention instances of healthcare-based discrimination, which significantly erode their trust in healthcare providers and institutions (Kaur et al., 2024). Furthermore, the power dynamics that operate in patient–provider interactions play a key role in the healthcare experience and rights awareness. A critical analysis pointed out that healthcare systems, while officially promoting charters of patient rights, may use institutional logics and practices that reduce formal civil rights to ‘patient experience problems’ (Kirkland & Hyman, 2021).

### Theoretical and practical implications

Our results suggest several theoretical and practical implications, as well as recommendations for improving the obstetric/gynaecological care experience. Better respect of patient rights, notably in the areas of consent, intimacy, and confidentiality, would be paramount. Our results tend to show that the effectiveness of the law is not optimal. patients' experiences of care do not correspond to the rights they enjoy in Canadian health care, as defined by charters of rights, more specifically in regard to consent, the right to obtain information, and the right to refuse treatments. Further studies are needed to better understand the legal and ethical frameworks associated with reproductive health care, both from the perspectives of those who receive the services and those who provide them. A more in-depth understanding of the effectiveness of the law in gynecological and obstetric care would also allow for more adequate information to be provided to patients who feel they have been wronged.

Respect of rights was shown to significantly improve satisfaction and trust in healthcare providers (Mahmoudi et al., 2017). According to the Person-Centered communication theory and the Montreal model in line with our findings, mechanisms to encourage trust, positive interactions, and empathy could also promote patient satisfaction (Lan & Yan, 2017). In addition, involving patients more actively in treatment decision-making would increase their satisfaction and feelings of empowerment, and hence increase their trust in the healthcare system (Cote, 2024; Wang et al., 2024). Implementing these measures would require mobilizing policy deciders and institutional managers to ensure that standards of practice prioritize patients' rights.

### Limitations

This study includes certain limitations that should be mentioned. Data were collected from a self-report questionnaire, which can introduce social desirability biases that influence participants’ responses. In addition, the reported care occurred within the seven years preceding data collection, which may also affect participants’ ability to accurately recall their experiences. Moreover, the sample, while substantial, was recruited mainly via social networks and partner organizations, which could introduce selection bias against satisfaction with obstetric/gynaecological treatment. Furthermore, the sample is not representative of the overall Québec population that has received obstetric/gynaecological care, notably in terms of age (few participants older than 56 years), education level (a relatively well-educated sample), or country of birth (overrepresentation of Canadian-born individuals). Furthermore, the majority of participants identified as cisgender women, which limits the generalization of the findings and the applicability of the conclusions to the experiences of trans men and nonbinary individuals. Finally, the absence of detailed qualitative data on the healthcare experiences precludes a deeper, nuanced, and contextual understanding of the dynamics that influenced the reported experiences.

Despite these limitations, this study has some noteworthy strengths. It is one of the first Québec studies to document recent experiences of obstetric/gynaecological care using a quantitative analysis combined with a participatory methodology that integrates community partners. The large and diversified sample, with individuals from diverse demographic groups, allows valuable insights into the care that is received in Québec’s healthcare system. Furthermore, the use of validated tools that are adapted to the local context thanks to a collaborative process, including the input of experts and other interested parties, confers considerable credibility and relevance on this study. Finally, the statistical analyses clearly determine associations between respect of rights, met needs regarding shared decision-making, and patient satisfaction. The results advance the knowledge and suggest approaches to improve practices, the organization of care, and healthcare policies.

## Conclusions

Our results open up several avenues for future research. Qualitative studies could delve deeper into individual narratives to better understand the underlying mechanisms of rights violations and dissatisfactory experiences, while exploring the dynamics that are specific to marginalized populations such as trans, nonbinary, and racialized individuals and residents in remote regions. Longitudinal studies could examine the long-term impact of interactions with healthcare providers and respect of rights on mental health, trust in the healthcare system, and subsequent medical consultations. Lastly, intervention studies could assess whether training programs centred on informed consent, an intercultural approach, and respect of patient rights can improve patients' experiences and satisfaction. These works could inform systemic reforms and help promote healthcare practices that are more equitable and respectful of the needs and rights of all patients.

## Data Availability

Since the data contain potentially sensitive information about study participants, the Human Research Ethics Board has only approved storage of the dataset on secure institutional servers. Any requests to access the data can be made to the first author or ciereh@uqam.ca.

## Endnote

1. The RAMQ is the provincial government organization responsible for administering Québec’s health insurance system and prescription drug insurance plan.

## References

[cit0001] Adams, N., Jacobsen, K., Li, L., Francino, M., Rutherford, L., Tei, C., Scheim, A., & Bauer, G. (2024). Health and health care access of autistic transgender and nonbinary people in Canada: A cross-sectional study. *Autism in Adulthood*, *7*(1), 66–80. 10.1089/aut.2023.0024 PMC11937772 40151657

[cit0002] Akbas, M. (2019). Patient satisfaction on nursing care: The case of gynecology and obstetrics clinics. *Acta Bioethica*, *25*(1), 127–136. 10.4067/S1726-569X2019000100127

[cit0003] Aoude, M. (2023). Factors that impact patient satisfaction and perceptions in patient-physician interactions in Canada: A literature review. *Undergraduate Research in Natural and Clinical Science and Technology Journal*, *7*, 1–9. 10.26685/urncst.430

[cit0004] Aranda, Z., Caamal, V., Montaño, M., Bernal, D., & Meneses, S. (2024). Exploring how non-clinical factors in childbirth care shape users’ experiences in public health facilities in rural Chiapas, Mexico: A qualitative study using the WHO health systems responsiveness framework. *BMC Pregnancy and Childbirth*, *24*(1), 1–14. 10.1186/s12884-024-06357-7 38424565 PMC10905866

[cit0005] Attanasio, L. B., McPherson, M. E., & Kozhimannil, K. B. (2014). Positive childbirth experiences in US hospitals: A mixed methods analysis. *Maternal and Child Health Journal*, *18*(5), 1280–1290. 10.1007/s10995-013-1363-1 24072597 PMC3966989

[cit0006] Aydin, E., Glasgow, K. A., Weiss, S. M., Khan, Z., Austin, T., Johnson, M. H., Barlow, J., & Lloyd-Fox, S. (2022). Giving birth in a pandemic: Women’s birth experiences in England during COVID-19. *BMC Pregnancy and Childbirth*, *22*(1), 304. 10.1186/s12884-022-04637-8 35399066 PMC8994823

[cit0007] Bhuiyan, J., & Loder, C. (2024). Evaluating patient experiences with patient-centered and inclusive care in academic obstetrics and gynecology outpatient clinics. *Journal of Patient Experience*, *11*, 23743735241297620. 10.1177/23743735241297620 39583039 PMC11583494

[cit0008] Birhanu, Z., Assefa, T., Woldie, M., & Morankar, S. (2010). Determinants of satisfaction with health care provider interactions at health centres in central Ethiopia: A cross sectional study. *BMC Health Services Research*, *10*, 78. 10.1186/1472-6963-10-78 20334649 PMC2848139

[cit0009] Bohren, M. A., Tunçalp, Ö., & Miller, S. (2020). Transforming intrapartum care: Respectful maternity care. *Best Practice & Research Clinical Obstetrics & Gynaecology*, *67*, 113–126. 10.1016/j.bpobgyn.2020.02.005 32245630

[cit0010] Bohren, M. A., Vogel, J. P., Hunter, E. C., Lutsiv, O., Makh, S. K., Souza, J. P., Aguiar, C., Saraiva Coneglian, F., Diniz, A. L. A., Tunçalp, Ö., Javadi, D., Oladapo, O. T., Khosla, R., Hindin, M. J., & Gülmezoglu, A. M. (2015). The mistreatment of women during childbirth in health facilities globally: A mixed-methods systematic review. *PLOS Medicine*, *12*(6), e1001847. 10.1371/journal.pmed.1001847 26126110 PMC4488322

[cit0011] Brown, H. L. (2017). Quality and safety in obstetrics and gynecology. *Clinical Obstetrics & Gynecology*, *60*(4), 818–828. 10.1097/grf.0000000000000324 28990984

[cit0012] Bohren, M. A., Mehrtash, H., Fawole, B., Maung, T. M., Balde, M. D., Maya, E., Thwin, S. S., Aderoba, A. K., Vogel, J. P., Irinyenikan, T. A., Adeyanju, A. O., Mon, N. O., Adu-Bonsaffoh, K., Landoulsi, S., Guure, C., Adanu, R., Diallo, B. A., Gülmezoglu, A. M., Soumah, A.-M., … Sall, A. O. (2019). How women are treated during facility-based childbirth in four countries: A cross-sectional study with labour observations and community-based surveys. *The Lancet*, *394*(10210), 1750–1763. 10.1016/S0140-6736(19)31992-0 PMC6853169 31604660

[cit0013] Cai, D., Villanueva, P., Lu, H., Zimmermann, B., & Horsch, A. (2024). What matters to migrant women during labor and birth: Chinese mothers’ experiences in Switzerland. *BMC Pregnancy and Childbirth*, *24*(1), 1–12. 10.1186/s12884-024-06271-y 38245713 PMC10799396

[cit0014] Cárdenas-Castro, M., & Salinero-Rates, S. (2023). The continuum of violence against women: Gynecological violence within the medical model in Chile. *Sexual & Reproductive Healthcare*, *37*, 100891. 10.1016/j.srhc.2023.100891 37480836

[cit0015] Chancogne, Z., & Lévesque, S. (2024). «À chaque fois que j’y allais, c’était comme partir à la bataille» : Une exploration qualitative des violences gynécologique et obstétricale au Québec. *Recherches Féministes*, *37*(1), 181–201. 10.7202/1114141ar

[cit0016] Cohen, J. E. (1988). *Statistical Power Analysis for the Behavioral Sciences*. Lawrence Erlbaum Associates, Inc.

[cit0017] Cote, C. I. (2024). A critical and systematic literature review of epistemic justice applied to healthcare: Recommendations for a patient partnership approach. *Medicine, Health Care, and Philosophy*, *27*(3), 455–477. 10.1007/s11019-024-10210-1 38833134

[cit0018] Cunningham, F. G., Leveno, K. J., Bloom, S. L., Spong, C. Y., Dashe, J. S., Hoffman, B. L., Casey, B. M., & Sheffield, J. S. (2014). *Williams Obstetrics* (Vol. 7) New York: McGraw-Hill Medical.

[cit0019] Di Meglio, G., & Yorke, E. (2019). L’accès universel à la contraception sans frais pour les jeunes du Canada. *Paediatrics & Child Health*, *24*(3), 165–169. 10.1093/pch/pxz047 PMC6519616 31110456

[cit0020] Dixon-Woods, M., Williams, S., Jackson, C., Akkad, A., Kenyon, S., & Habiba, M. (2006). Why do women consent to surgery, even when they do not want to? An interactionist and Bourdieusian analysis. *Social Science & Medicine*, *62*(11), 2742–2753. 10.1016/J.SOCSCIMED.2005.11.006 16343723

[cit0021] Engeltjes, B., van Herk, N., Visser, M., van Wijk, A., Cronie, D., Rosman, A., Scheele, F., & Wouters, E. (2023). Patients' experiences with an obstetric telephone triage system: A qualitative study. *Patient Education and Counseling*, *108*, 1–7. 10.1016/j.pec.2022.107610 36584556

[cit0022] Epstein, R. M., Franks, P., Fiscella, K., Shields, C. G., Meldrum, S. C., Kravitz, R. L., & Duberstein, P. R. (2005). Measuring patient-centered communication in patient-physician consultations: Theoretical and practical issues. *Social Science & Medicine (1982)*, *61*(7), 1516–1528. 10.1016/j.socscimed.2005.02.001 16005784

[cit0023] Flora, L., Pomey, M.-P., Karazivan, P., Dumez, V., Lebel, P., Vanier, M.-C., Débarges, B., Clavel, N., & Jouet, E. (2015). Le « Montreal model »: enjeux du partenariat relationnel entre patients et professionnels de la santé. *Santé Publique*, *S1*(HS), 41–50. 10.3917/spub.150.0041 26168616

[cit0024] Gaucher, L., Huissoud, C., Ecochard, R., Rudigoz, R. C., Cortet, M., Bouvet, L., Bouveret, L., Touzet, S., Gonnaud, F., Colin, C., Gaucherand, P., Dupont, C., & Group, A. (2021). Women's dissatisfaction with inappropriate behavior by health care workers during childbirth care in France: A survey study. *Birth*, *48*(3), 328–337. 10.1111/birt.12542 33686732

[cit0025] Gauthier, J., Haggerty, J., Lamarche, P., Lévesque, J.-F., Morin, D., Pineault, R., Hamel, M., Lessard, L., Sylvain, H., Perron, M., & Roberge, D. (2009). Entre adaptabilité et fragilité: les conditions d'accès aux services de santé des communautés rurales et éloignées. *Institut National de Santé Publique du Québec*, 58. https://www.inspq.qc.ca/publications/1014

[cit0026] Ghabrial, M. A., Ferguson, T., Scheim, A. I., Adams, N. J., Khatoon, M., & Bauer, G. R. (2024). Factors associated with primary healthcare provider access among trans and non-binary immigrants, refugees, and newcomers in Canada. *Journal of Migration and Health*, *10*, 100241. 10.1016/j.jmh.2024.100241 39040891 PMC11261874

[cit0027] Gouvernement du Québec. (2018). *Lieux pour l'accouchement- Services de Sage-femme*. https://www.quebec.ca/sante/systeme-et-services-de-sante/organisation-des-services/services-de-sage-femme/lieux-pour-l-accouchement

[cit0028] Government of Canada, Library of Parliament. (2019). The Canada Health Act: An Overview, publication No 2-19-54-E (p. 18).

[cit0029] Government of Canada. (2024). *Canada's Health Care System*. https://www.canada.ca/en/health-canada/services/health-care-system/reports-publications/health-care-system/canada.html

[cit0030] Haywood, C., Lanzkron, S., Bediako, S., Strouse, J., Haythornthwaite, J., Carroll, C., Diener-West, M., Onojobi, G., Beach, M., & Investigators, F. (2014). Perceived discrimination, patient trust, and adherence to medical recommendations among persons with sickle cell disease. *Journal of General Internal Medicine*, *29*, 1657–1662. 10.1007/s11606-014-2986-7 25205621 PMC4242876

[cit0031] Kaiser, H. F. (1960). The application of electronic computers to factor analysis. *Educational and Psychological Measurement*, *20*(1), 141–151. 10.1177/001316446002000116

[cit0032] Kaur, A., Gottlieb, L., De Cuba, S., Byhoff, E., Fleegler, E., Cohen, A., Glasser, N., Ommerborn, M., Clark, C., & De Marchis, E. (2024). Associations between patient/caregiver trust in clinicians and experiences of healthcare-based discrimination. *Journal of the American Board of Family Medicine*, *37*(4), 607–636. 10.3122/jabfm.2023.230182r1 39455263

[cit0033] Kirkland, A., & Hyman, M. (2021). Civil rights as patient experience: How healthcare organizations handle discrimination complaints. *Law & Society Review*, *55*, 273–295. 10.1111/LASR.12554

[cit0034] Kowalewska, B., Kamińska, A., Rolka, H., Ortman, E., & Krajewska–Kułak, E. (2014). Satisfaction with obstetric care in the early postnatal period. *Progress in Health Sciences*, *4*(1), 95–101.

[cit0035] Lan, Y., & Yan, Y. (2017). The impact of trust, interaction, and empathy in doctor-patient relationship on patient satisfaction. *Journal of Nursing and Health*, *2*(2-8), 1–8. 10.21767/2574-2825.100015

[cit0036] Lévesque, S., Manon, B., Fontaine, L., Rousseau, C., & Beauchemin-Roy, S. (2016). Détresse, souffrance ou violence obstétricale lors de l’accouchement: Perspectives des intervenantes communautaires membres. *du Regroupement Naissance-Renaissance*, 82. 10.13140/RG.2.2.33053.51682

[cit0037] Mahmoudi, G., Mahmoudvand, H., Adeli, M., Taghadosi, A., & Bahmani, M. (2017). Evaluation of respect for patient's rights from the viewpoint of hospitalized surgical patinents in Shohada Ashayer Hospital of Khorramabad City, Iran in 2015-2016. *International Journal of Current Pharmaceutical Review and Research*, *8*(1), 49–52. 10.25258/ijcprr.v8i01.9089

[cit0038] McCrea, B. H., & Wright, M. E. (1999). Satisfaction in childbirth and perceptions of personal control in pain relief during labour. *Journal of Advanced Nursing*, *29*(4), 877–884. 10.1046/j.1365-2648.1999.00961.x 10215979

[cit0039] Prall, S., Scelza, B., & Davis, H. E. (2024). Medical mistrust, discrimination and healthcare experiences in a rural Namibian Community. *Global Public Health*, *19*(1), 1–13. 10.1080/17441692.2024.2346207 38718288

[cit0040] Qaderi, K., Geranmayeh, M., Farnam, F., Sheikh Hasani, S., & Mirmolaei, S. T. (2021). Understanding HPV-positive women’s needs and experiences in relation to patient-provider communication issues: A qualitative study. *BMC Health Services Research*, *21*(1), 286. 10.1186/s12913-021-06283-w 33784992 PMC8011207

[cit0041] Redshaw, M., Martin, C. R., Savage-McGlynn, E., & Harrison, S. (2019). Women’s experiences of maternity care in England: Preliminary development of a standard measure. *BMC Pregnancy and Childbirth*, *19*(1), 1–13. 10.1186/s12884-019-2284-9 31088487 PMC6518811

[cit0042] Scheim, A. I., Coleman, T., Lachowsky, N., & Bauer, G. R. (2021). Health care access among transgender and nonbinary people in Canada, 2019: A cross-sectional survey. *CMAJ Open*, *9*(4), E1213–E1222. 10.9778/cmajo.20210061 PMC8695530 34933879

[cit0043] Shalowitz, D., & Ralston, S. (2023). Safeguards for procedural consent in obstetric care. *Journal of Medical Ethics*, *49*, 628–629. 10.1136/jme-2023-109212 37344200

[cit0044] Toupin, A., & Lévesque, S. (2025). Experiences and perceptions of gynaecological violence: A descriptive exploration of the phenomenon from survivors’ standpoints. *Feminist Encounters: A Journal of Critical Studies in Culture and Politics*, *9*(1), 1–17. 10.20897/femenc/16022

[cit0045] Trainor, L., Frickberg-Middleton, E., McLemore, M., & Franck, L. (2020). Mexican-born women’s experiences of perinatal care in the United States. *Journal of Patient Experience*, *7*(6), 941–945. 10.1177/2374373520966818 33457525 PMC7786653

[cit0046] Vargas, B., Louzado-Feliciano, P., Santos, N., Fuller, S., Jimsheleishvili, S., Quiñones, Á., & Martin, H. H. (2021). An exploration of patient-provider dynamics and childbirth experiences in rural and urban Peru: A qualitative study. *BMC Pregnancy and Childbirth*, *21*(1), 1–15. 10.1186/s12884-021-03586-y 33588780 PMC7885576

[cit0047] Vedam, S., Stoll, K., Rubashkin, N., Martin, K., Miller-Vedam, Z., Hayes-Klein, H., & Jolicoeur, G. (2017). The mothers on respect (MOR) index: Measuring quality, safety, and human rights in childbirth. *SSM - Population Health*, *3*, 201–210. 10.1016/j.ssmph.2017.01.005 29349217 PMC5768993

[cit0048] Vedam, S., Stoll, K., Taiwo, T. K., Rubashkin, N., Cheyney, M., Strauss, N., McLemore, M., Cadena, M., Nethery, E., Rushton, E., Schummers, L., Declercq, E., & the, G.-U. S. S. C. (2019). The giving voice to mothers study: Inequity and mistreatment during pregnancy and childbirth in the United States. *Reproductive Health*, *16*(1), 77. 10.1186/s12978-019-0729-2 31182118 PMC6558766

[cit0049] Wang, H., Jia, J., Fan, Y., Chen, H., Lou, Y., Wang, X., & Huang, X. (2024). Impact of inpatient self-efficacy and trust in physicians on inpatient satisfaction with medical services: the mediating role of patient participation in medical decision-making. *Frontiers in Psychology*, *15*, 1364319. 10.3389/fpsyg.2024.1364319 39282672 PMC11392843

[cit0050] Worabo, H. J., Safi, F., Gill, S. L., & Farokhi, M. (2024). “It’s different here” Afghan refugee maternal health experiences in the United States. *BMC Pregnancy and Childbirth*, *24*(1), 1–12. 10.1186/s12884-024-06678-7 39014313 PMC11251342

[cit0051] Wylie, L., McConkey, S., & Corrado, A. M. (2021). It’s a journey not a check box: Indigenous cultural safety from training to transformation. *International Journal of Indigenous Health*, *16*(1), 314–332. 10.32799/ijih.v16i1.33240

